# BCL-W makes only minor contributions to MYC-driven lymphoma development

**DOI:** 10.1038/s41388-023-02804-5

**Published:** 2023-08-11

**Authors:** Sarah T. Diepstraten, John E. La Marca, Catherine Chang, Savannah Young, Andreas Strasser, Gemma L. Kelly

**Affiliations:** 1 Blood Cells and Blood Cancer Division, The Walter and Eliza Hall Institute of Medical Research (WEHI), Parkville, VIC Australia; 2 Department of Medical Biology, University of Melbourne, Parkville, VIC Australia

**Keywords:** B-cell lymphoma, Biochemistry

## Abstract

The BH3-mimetic drug Venetoclax, a specific inhibitor of anti-apoptotic BCL-2, has had clinical success for the treatment of chronic lymphocytic leukaemia and acute myeloid leukaemia. Attention has now shifted towards related pro-survival BCL-2 family members, hypothesising that new BH3-mimetic drugs targeting these proteins may emulate the success of Venetoclax. BH3-mimetics targeting pro-survival MCL-1 or BCL-XL have entered clinical trials, but managing on-target toxicities is challenging. While increasing evidence suggests BFL-1/A1 is a resistance factor for diverse chemotherapeutic agents and BH3-mimetic drugs in haematological malignancies, few studies have explored the role of BCL-W in the development, expansion, and therapeutic responses of cancer. Previously, we found that *BCL-W* was not required for the ongoing survival and growth of various established human Burkitt lymphoma and diffuse large B cell lymphoma cell lines. However, questions remained about whether BCL-W impacts lymphoma development. Here, we show that BCL-W appears dispensable for MYC-driven lymphomagenesis, and such tumours arising in the absence of BCL-W show no compensatory changes to BCL-2 family member expression, nor altered sensitivity to BH3-mimetic drugs. These results demonstrate that BCL-W does not play a major role in the development of MYC-driven lymphoma or the responses of these tumours to anti-cancer agents.

## Introduction

Apoptosis is a process of regulated cell death, where signals triggered by intrinsic or extrinsic factors result in the ordered destruction of cells. It is critical for embryonic development, maintenance of tissue homoeostasis, and the removal of dispensable or potentially dangerous (e.g. infected) cells [1]. In mammals, induction of the intrinsic apoptosis pathway proceeds via upregulation of the pro-apoptotic BH3-only proteins (e.g. BIM, PUMA, NOXA). For some BH3-only proteins, such as PUMA and NOXA, this can occur directly through the master regulator TP53, a transcription factor activated by cellular stresses like DNA damage [2–4]. These BH3-only proteins can then bind and inhibit the pro-survival BCL-2 family members: BCL-2, BCL-XL, MCL-1, BFL-1/A1, and BCL-W. This liberates the pro-apoptotic effectors BAK and BAX to execute cell death by causing mitochondrial outer membrane permeabilization (MOMP), unleashing a cascade of caspases for demolition of the cell [5].

Abnormally increased expression of pro-survival BCL-2 proteins or decreased levels of the pro-apoptotic BCL-2 family members have been implicated in the development and therapy resistance of a variety of cancers, particularly leukaemias and lymphomas [6]. The development of BH3-mimetics, drugs capable of directly binding and inhibiting select pro-survival BCL-2 family members, has led to a paradigm shift in how certain cancers are treated and how drugs are designed [7]. ABT-737, the first BH3-mimetic drug developed, binds to BCL-2, BCL-XL, and BCL-W [8]. It was soon superseded by ABT-263/Navitoclax, which has identical specificity but is orally available [9]. Clinical deployment of ABT-263/Navitoclax has been challenging due to BCL-XL being required for platelet survival [10, 11], though chemically conjugating the BCL-XL-specific BH3-mimetic AZD4320 with a PEGylated poly-lysine dendrimer improved its targeting to the lymphatic system, thereby reducing on-target platelet toxicity [12]. The most clinically advanced BH3-mimetic drug is Venetoclax, which selectively targets BCL-2 and is approved by several regulatory bodies worldwide for the treatment of chronic lymphocytic leukaemia (CLL) and acute myeloid leukaemia (AML) [13]. However, many cancers depend on different pro-survival proteins for sustained growth (e.g. MCL-1) and so do not respond to Venetoclax. Moreover, acquired resistance to Venetoclax can occur through the upregulation of non-targeted pro-survival BCL-2 proteins, such as MCL-1 or BFL-1 [14, 15]. This has spurred interest towards developing BH3-mimetic drugs targeting other pro-survival BCL-2 proteins. MCL-1 is known to be important for the sustained growth of several cancers and BH3-mimetic drugs targeting MCL-1 are currently in clinical trials for select blood cancers [16]. However, BCL-W is considerably less well-studied, and any roles of this pro-survival protein in tumour growth are not well understood.

BCL-W, encoded by the BCL2 like 2 (*BCL2L2*) gene, was first identified as a pro-survival protein in lymphoid and myeloid cells [17]. However, only spermatogenesis-related cells have thus far been identified to rely on BCL-W for their survival [18–20]. Consequently, BCL-W-deficient male mice are sterile, but otherwise both genders develop and age normally [18–20]. Due to this limited necessity, there is considerable interest in determining whether BCL-W is required for the survival of cancer cells, and thus might be an attractive target for the development of specific BH3-mimetic drugs. BCL-W expression is upregulated in certain cancer samples relative to normal tissues, such as some digestive tract malignancies [21, 22], as well as some Burkitt lymphomas (BLs), diffuse large B cell lymphomas (DLBCLs), and Hodgkin lymphomas (HLs) [23, 24]. However, debate persists over whether any cancer cells, particularly haematopoietic malignancies, rely specifically on BCL-W for their sustained survival. It has been reported that the absence of BCL-W induces apoptosis in human BL cell lines [25]; however, we found that BCL-W is dispensable for the ongoing survival of human BL and DLBCL cell lines, whether non-stressed, suffering from nutrient deprivation, or undergoing treatment with BH3-mimetic drugs [26].

There are also questions about whether BCL-W is necessary for the development of haematological malignancies. For example, BCL-W overexpression has been shown to accelerate development of MYC-driven myeloid leukaemia [27], but it has also been shown to be dispensable for AML development [28]. Furthermore, BCL-W has recently been shown to significantly extend the lifespan of mice carrying the *Eµ-Myc* transgene [25], a powerful driver of pre-B/B cell lymphomagenesis. In the present study, we also investigated the role of BCL-W in the development of MYC-driven B cell lymphoma. We found that the absence of BCL-W did not have significant impact on the rate and severity of pre-B/B lymphoma development in *Eµ-Myc* transgenic mice. Compared to control *Eµ-Myc* lymphomas, those arising in BCL-W-deficient *Eµ-Myc* mice did not display any notable differences in their immunophenotype, expression of apoptosis-related proteins, or responses to BH3-mimetic drugs. These findings, in combination with our previous work, indicate BCL-W does not have a major role in either the development or continued survival of MYC-driven B cell lymphomas, and therefore is not an attractive therapeutic target for these malignancies.

## Results

### The absence of BCL-W has only marginal impact on lymphoma development in *Eµ-Myc* mice

**Fig. 1:**
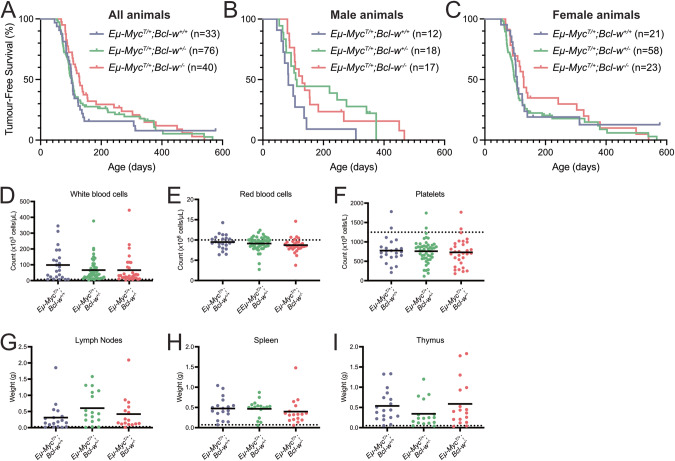
The absence of BCL-W does not markedly impact the survival of *Eµ-Myc* transgenic mice. **A**–**C** Kaplan-Meier survival curves representing the tumour-free survival of all *Eµ-Myc*^*T/+*^*;Bcl-w*^*+/+*^, *Eµ-Myc*^*T/+*^*;Bcl-w*^*+/−*^, or *Eµ-Myc*^*T/+*^*;Bcl-w*^−*/*−^ mice (**A**), or stratified by gender (**B**, **C**). No significant differences, regardless of gender stratification, were observed when comparing *Eµ-Myc*^*T/+*^*;Bcl-w*^*+/+*^ animals to either *Eµ-Myc*^*T/+*^*;Bcl-w*^*+/−*^ or *Eµ-Myc*^*T/+*^*;Bcl-w*^*−/−*^ animals (Mantel-Cox tests with Bonferonni’s multiple comparisons (K = 2), adjusted *p* > 0.025). **D**–**F** Quantification of blood cell types in moribund *Eµ-Myc*^*T/+*^*;Bcl-w*^*+/+*^, *Eµ-Myc*^*T/+*^*;Bcl-w*^*+/*−^, and *Eµ-Myc*^*T/+*^*;Bcl-w*^*−/*−^ mice. No significant differences were observed between the genotypes for either white blood cells (**D**), red blood cells (**E**), or platelets (**F**). **G**–**I** Quantification of organ weights from sick *Eµ-Myc*^*T/+*^*;Bcl-w*^*+/+*^, *Eµ-Myc*^*T/+*^*;Bcl-w*^*+/*−^, and *Eµ-Myc*^*T/+*^*;Bcl-w*^−*/*−^ mice at ethical endpoint. No significant differences were observed between the genotypes for either the lymph nodes (**G**), spleen (**H**), or thymus (**I**). In each graph, the dotted line represents the average result for that parameter from healthy C57BL/6 mice.

To investigate whether BCL-W has a role in MYC-driven lymphomagenesis, we generated *Eµ-Myc*^*T/+*^*;Bcl-w*^*+/−*^ male mice, and then crossed them with *Bcl-w*^*+/−*^ or *Bcl-w*^*−/*−^ females. There were no statistically significant differences in tumour latency between the *Eµ-Myc*^*T/+*^*;Bcl-w*^*+/+*^ animals and either the *Eµ-Myc*^*T/+*^*;Bcl-w*^*+/*−^ or *Eµ-Myc*^*T/+*^*;Bcl-w*^*−/−*^ animals (Mantel-Cox test with Bonferonni’s multiple comparisons (K = 2), adjusted *p* > 0.025) (Fig. 1A).

Separating the animals by gender suggested males diverged somewhat from the population at large. However, separating males and females and comparing tumour-free survival between the *Eµ-Myc*^*T/+*^*;Bcl-w*^*+/+*^ animals and either the *Eµ-Myc*^*T/+*^*;Bcl-w*^*+/*−^ or *Eµ-Myc*^*T/+*^*;Bcl-w*^*−/−*^ animals still did not reveal any significant differences (Mantel-Cox tests with Bonferonni’s multiple comparisons (K = 2), adjusted *p* > 0.025) (Fig. 1B, C). However, there were larger differences in male survival times (Fig. 1B, Table 1), likely partly due to several *Eµ-Myc*^*T/+*^*;Bcl-w*^*−/*−^ animals that persisted beyond 200 days (*n* = 4/17). This suggests any small effect *Bcl-w* loss might be having is not a consistent, population-wide effect, but rather restricted to a small number of outlier animals. Together, these data indicate that *Bcl-w* loss has no impact on the lymphoma-free survival of *Eµ-Myc* mice.

**Table 1:** Median tumour-free survival times for each genotype and gender.

Median tumour-free survival (days)			
	*Eµ-Myc*^*T/+*^*; Bcl-w*^*+/+*^	*Eµ-Myc*^*T/+*^*; Bcl-w*^*+/−*^	*Eµ-Myc*^*T/+*^*; Bcl-w*^−*/−*^
Combined males and females	102	103.5	128
Males	92	110	130
Females	106	98	128

Small differences in survival were observed, particularly in the male mice, but these differences did not result in statistically significant differences between the genotypes, regardless of gender. Tumour free-survival time is measured in days.

As expected [29], lymphoma-burdened *Eµ-Myc* mice had increased white blood cell counts compared to healthy wild-type controls, and their red blood cell and platelet counts were decreased (Fig. 1D–F). No significant differences in any of these parameters were observed between lymphoma-burdened *Eµ-Myc*^*T/+*^*;Bcl-w*^*−/−*^ and control *Eµ-Myc*^*T/+*^*;Bcl-w*^*+/+*^ mice at ethical end point. Sick *Eµ-Myc* mice typically present with enlarged lymph nodes, spleens, and thymii due to their lymphoma burden [29]. No significant differences in the weights of any of these tissues were observed between *Eµ-Myc*^*T/+*^*;Bcl-w*^*−/*−^ and control *Eµ-Myc*^*T/+*^*;Bcl-w*^*+/+*^ mice at ethical end point (Fig. 1G–I).

### The absence of BCL-W does not cause a change in the immunophenotype of *Eµ-Myc* lymphomas

**Fig. 2:**
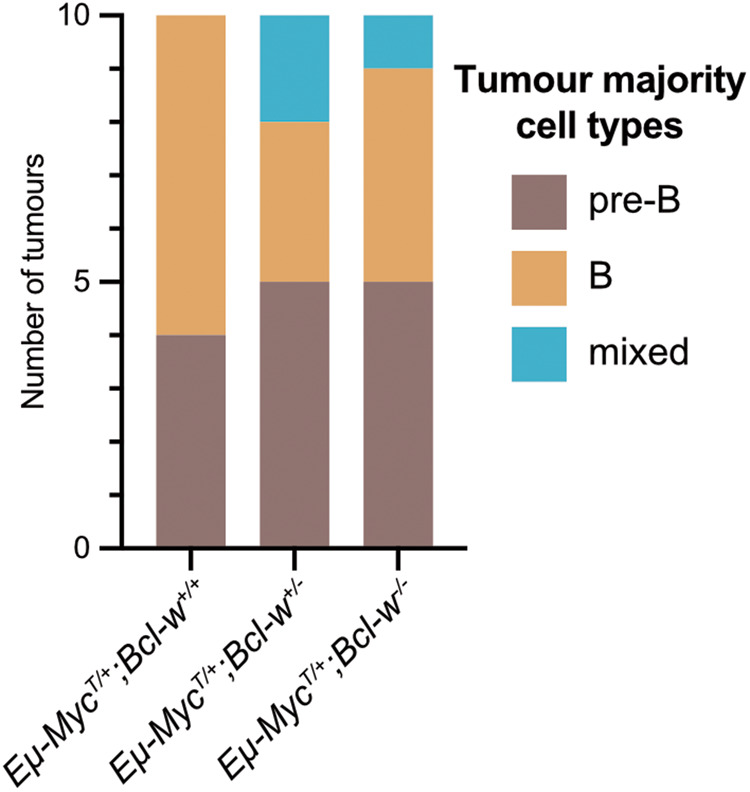
Absence of BCL-W has no obvious impact on the immunophenotype of MYC-driven B cell lymphomas. Immunophenotyping results from lymphoma-burdened lymph nodes or spleens of 10 of each of *Eµ-Myc*^*T/+*^*;Bcl-w*^*+/+*^, *Eµ-Myc*^*T/+*^*;Bcl-w*^*+/−*^, or *Eµ-Myc*^*T/+*^*;Bcl-w*^*−/*−^ mice. The majority cell populations (pre-B (B220^+^IgM^-^IgD^-^) or B (B220^+^IgM^+^IgD^-^ or B220^+^IgM^+^IgD^+^)) constituting the malignant tissues were quantified by flow cytometry, and the lymphomas were then categorised on that basis. Overall, there were only minimal difference between lymphomas of the different genotypes, apart from a lack mixed pre-B/ B cell lymphomas in *Eµ-Myc*^*T/+*^*;Bcl-w*^*+/+*^ animals.

Lymphomas from *Eµ-Myc*^*T/+*^*;Bcl-w*^*+/+*^, *Eµ-Myc*^*T/+*^*;Bcl-w*^*+/−*^, or *Eµ-Myc*^*T/+*^*;Bcl-w*^*−/−*^ mice were immunophenotyped by staining for the B cell lineage surface markers IgM, IgD, CD19, and B220 (B220^+^IgM^−^IgD^−^ identifies pro-B/pre-B cells, B220^+^IgM^+^IgD^−^ identifies immature B cells, and B220^+^IgM^+^IgD^+^ identifies mature B cells). Lymphomas with a mature B cell phenotype were more common in *Eµ-Myc*^*T/+*^*;Bcl-w*^*+/+*^ mice, while lymphomas from both *Eµ-Myc*^*T/+*^*;Bcl-w*^*+/*−^ and *Eµ-Myc*^*T/+*^*;Bcl-w*^−*/*−^ animals occasionally presented with a mixed pre-B/B cell immunophenotype (Fig. 2). However, there was generally no clear difference in immunophenotype of lymphomas between mice of these three genotypes.

### Lymphomas from *Eµ-Myc*^*T/+*^*;Bcl-w*^*−/−*^ mice do not show marked compensatory changes in the expression of members of the BCL-2 protein family

**Fig. 3:**
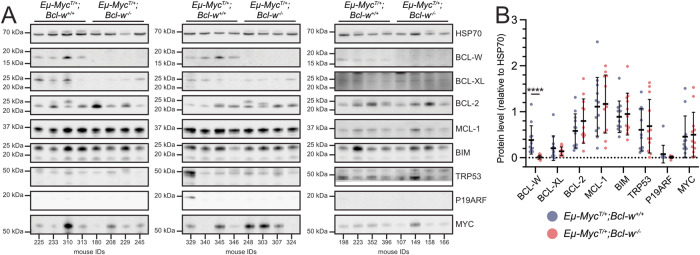
The absence of BCL-W does not result in compensatory upregulation of other pro-survival BCL-2 family members in MYC-driven lymphomas. **A** Western blots examining and quantifying the expression levels of different BCL-2 family members and other regulators of the intrinsic apoptosis pathway. 12 lymphoma samples from spleens from each of *Eµ-Myc*^*T/+*^*;Bcl-w*^*+/+*^ and *Eµ-Myc*^*T/+*^*;Bcl-w*^*−/−*^ animals were examined, with male (*n* = 9) and female (*n* = 15) animals fairly represented (mouse IDs are listed along the bottom of each image (see Supplementary File 1 for complete cohort information)). Apart from BCL-W expression, no obvious, consistent differences in the levels of the indicated proteins were found between lymphomas of the two genotypes. Note that the HSP70 blots shown are representatives from one blot, as the experiment was undertaken over multiple blots, with HSP70 used as the loading control in each instance. Note also that the additional bands seen in the BCL-2 blots are due to these lymphomas being IgM-positive, as determined by flow cytometry; i.e. the bands detected are due to the binding of the HRP-conjugated anti-mouse-Ig secondary antibody to mouse Ig light chain. Complete blots are shown in Supplementary Fig. 1. **B** Quantification of the protein expression levels revealed that only BCL-W was differentially expressed between lymphomas of the two genotypes (Mann–Whitney test with FDR multiple comparisons, mean rank *Eµ-Myc*^*T/+*^*;Bcl-w*^*+/+*^ = 18.5 and *Eµ-Myc*^*T/+*^*;Bcl-w*^*−*^^*/−*^ = 6.5, U = 0.0, n(*Eµ-Myc*^*T/+*^*;Bcl-w*^*+/+*^) = n(*Eµ-Myc*^*T/+*^*;Bcl-w*^*−/−*^) = 12, *p* < 0.0001). Data in (**B**) are presented as mean ± standard deviation. **** = *p* < 0.0001. While all data are displayed on one graph, comparisons should not be made between different proteins, as apparent differences in expression may be due to variable blot exposure or differences in antibody binding affinities.

A frequent mechanism of resistance in malignant cells to loss/inhibition of BCL-2 pro-survival proteins by BH3-mimetic drugs is the upregulation of one/several of the non-targeted pro-survival BCL-2 family proteins [16, 30]. If BCL-W is required to support the ongoing survival of lymphoma cells, it is possible that such compensatory changes could occur to allow MYC-driven lymphomagenesis in the absence of BCL-W. To investigate this, we performed Western blot analyses on tumour tissues from *Eµ-Myc*^*T/+*^*;Bcl-w*^*+/+*^ and *Eµ-Myc*^*T/+*^*;Bcl-w*^*−/−*^ mice (*n* = 12 per genotype; *n*(females) = 15, *n*(males) = 9) (Fig. 3A). As expected, no BCL-W was detected in lymphoma samples from *Eµ-Myc*^*T/+*^*;Bcl-w*^*−/−*^ mice, whereas low levels were found in tumours from *Eµ-Myc*^*T/+*^*;Bcl-w*^*+/+*^ mice (Fig. 3A, B). No marked differences in the other BCL-2 family proteins examined (BCL-XL, BCL-2, MCL-1, BIM) were observed between lymphomas of the two genotypes (Fig. 3A, B). Furthermore, high levels of TRP53 or P19ARF connote *TRP53* mutations in a tumour, and no consistent differences in the frequency of lymphomas with TRP53 defects were observed between lymphomas from *Eµ-Myc*^*T/+*^*;Bcl-w*^*+/+*^ and *Eµ-Myc*^*T/+*^*;Bcl-w*^*−/−*^ mice (Fig. 3A, B). Collectively, these data provide evidence that there are no marked compensatory changes in other BCL-2 family members, nor altered selection for defects in the tumour suppressor TRP53, when MYC-driven lymphomas develop in the absence of BCL-W.

### Cell lines generated from *Eµ-Myc*^*T/+*^*;Bcl-w*^*−/*−^ lymphomas respond similarly to BH3-mimetic drug treatment as those derived from control *Eµ-Myc*^*T/+*^ lymphomas

**Fig. 4:**
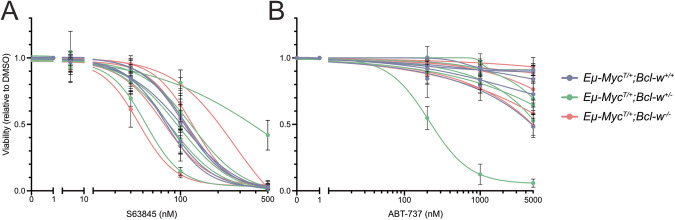
The absence of BCL-W has no impact on the survival of lymphoma-derived cell lines after treatment with BH3-mimetic drugs. **A**, **B** Results from independent lymphoma-derived cell lines from five of each of *Eµ-Myc*^*T/+*^*;Bcl-w*^*+/+*^, *Eµ-Myc*^*T/+*^*;Bcl-w*^*+/*−^, or *Eµ-Myc*^*T/+*^*;Bcl-w*^−*/*−^ mice, examined for their viability after treatment for 24 h with the indicated BH3-mimetic drugs. No differences in viability were observed between lymphoma cell lines of the different genotypes, regardless of whether (**A**) S63845 (inhibitor of MCL-1) or (**B**) ABT-737 (inhibitor of BCL-2 + BCL-XL + BCL-W) was used. In addition, while S63485 was able to robustly induce death in the lymphoma cells, these cells were largely resistant to treatment with ABT-737.

Next, we generated cell lines from lymphomas from *Eµ-Myc*^*T/+*^*;Bcl-w*^*+/+*^, *Eµ-Myc*^*T/+*^*;Bcl-w*^*+/−*^, and *Eµ-Myc*^*T/+*^*;Bcl-w*^−*/−*^ mice (*n* = 5 each) to examine whether MYC-driven lymphomas that had developed in the absence of BCL-W would respond differently to anti-cancer agents. These lymphoma lines were treated in culture with a range of concentrations of the BH3-mimetic drugs S63845 (MCL-1 inhibitor [31]) and ABT-737 (inhibitor of BCL-2 + BCL-XL + BCL-W [8]). If BCL-W was important for tumour cell survival, it may be expected that loss of BCL-W would sensitise the lymphomas to one/both of these BH3-mimetic drugs. Treatment with S63845 caused a marked decrease in cell viability, with no differences observed between lymphoma cells of the two genotypes (Fig. 4A). ABT-737 had little impact on the viability of lymphomas of either genotype (Fig. 4B). These findings show that the absence of BCL-W does not alter the sensitivity of lymphoma cells to BH3-mimetic drugs targeting other pro-survival BCL-2 proteins.

## Discussion

The BCL-2-specific BH3-mimetic drug Venetoclax is used for the treatment of patients with CLL and AML, and inhibitors of MCL-1 or BCL-XL have shown efficacy in pre-clinical models but are less advanced clinically [16]. Efforts towards developing inhibitors of the relatively understudied pro-survival BCL-2 family members, BCL-W and BFL-1/A1, are ongoing. However, there is significant debate over the importance of BCL-W for the development and sustained growth of cancers, particularly haematological malignancies. In this study, we found that the absence of BCL-W had no significant impact on MYC-driven lymphoma development in vivo. Moreover, the absence of BCL-W had no impact on severity of malignant disease, lymphoma immune-phenotype(s), expression of other BCL-2 family members or responses to inhibitors of MCL-1 or BCL-2 + BCL-XL + BCL-W.

The most striking difference between published data and ours are the lymphoma-free survival curves. It was previously reported that the absence of BCL-W substantially and significantly delayed lymphoma development in *Eµ-Myc* mice, with *Eµ-Myc*^*T/+*^*;Bcl-w*^*+/+*^ mice all having succumbed to lymphoma before the first *Eµ-Myc*^*T/+*^*;Bcl-w*^*−/−*^ mouse even fell ill, and the *Eµ-Myc*^*T/+*^*;Bcl-w*^*−/*−^ mice survived upwards of 3 times longer than the controls [25]. We did not observe a similarly impressive result in our mouse cohorts. How do we reconcile the differences between the two studies? Both studies used *Eµ-Myc* transgenic mice [32] derived from the same strain (though being maintained in separate locations some genetic drift has likely occurred), but the source of the *Bcl-w* knockout mutation differs: Print et al. [18] in this study vs Ross et al. [19] in the previous study. It is possible that whole genome sequencing might reveal whether epistatic mutations are present in the genomes of either of these *Bcl-w*^*−/−*^ strains. Furthermore, while not significant, we did observe a small increase in tumour-free survival in *Eµ-Myc*^*T/+*^*;Bcl-w*^*−/−*^ males, but this result appeared to be driven only by a small number of very old mice. Given the essential role of BCL-W in spermatogenesis, and the lack of other defects in *Bcl-w*^−/−^ mice [18–20], it may be tempting to speculate on a link between the production of male sex hormones and the minor delay in MYC-driven lymphoma development. This hypothesis could be tested by crossing *Eµ-Myc* mice with other mutant mice with spermatogenesis defects but no other abnormalities, such as *spermatid maturation 1* mice [33, 34].

It is interesting to compare the roles of the different BCL-2 family members in MYC-driven lymphoma development. Like our data for BCL-W, the absence of A1 had no impact on lymphoma development in *Eµ-Myc* mice [35]. The absence of BCL-2 also did not delay lymphoma development, and this is explained by the observation that its levels are very low in pre-B and immature B lymphocytes, the cells from which these lymphomas arise [36]. By contrast, the removal of BCL-XL or MCL-1 (even just one allele of *Mcl-1*) greatly delayed lymphoma development in *Eµ-Myc* mice [37, 38]. Of note, BCL-XL and MCL-1 are both expressed in pre-B and immature B lymphocytes and appear essential for the survival of these cells during oncogene-driven (e.g. MYC over-expression) neoplastic transformation.

Finally, in various solid cancers, BCL-W has been detected as a potential resistance factor [39], and is upregulated in a host of these malignancies [40], but to our knowledge this is not the case in haematological cancers. We recently published the results of a CRISPR activation (CRISPRa) screen in a murine model of double-hit lymphoma (DHL), where we identified resistance factors to Venetoclax treatment [30]. Interestingly, in this screen we identified upregulation of genes expressing the pro-survival BCL-2 family members BCL-XL, MCL-1, and A1 as factors that promoted DHL resistance to Venetoclax, but not BCL-W. While this screen was limited to DHL as the cancer model, as CRISPRa technology becomes more accessible it will be interesting to see whether increased expression of BCL-W will emerge as a survival-promoting factor in other cancer types, or in the face of other treatments.

To conclude, our present and previously published [26] data do not support the idea that BCL-W is an attractive therapeutic target for the treatment of B lymphoid malignancies.

## Materials and methods

### Animal studies

All studies using animals were conducted according to the WEHI Animal Ethics Committee guidelines and performed with approval of said committee. *Eµ-Myc* [32] and *Bcl-w* [18] knockout mice are as published. All mice used were on a C57BL/6-WEHI background. See supplementary materials for additional information.

### Western blotting

Frozen tumour single cell suspensions (from spleen) were thawed into FMA medium (high glucose DMEM supplemented with 10% heat-inactivated foetal bovine serum (FBS), 100 µM L-asparagine, and 50 µM β-mercapto-ethanol). Centrifuged cells were resuspended in radioimmunoprecipitation assay (RIPA) buffer (30 µL per 1 × 10^6^ cells), with protease inhibitors (Roche #11836145001). Protein concentration was measured using the Pierce BCA assay kit (Thermo Fisher #23225). 20 µg of protein was loaded per well. Primary and HRP-conjugated secondary antibodies are listed in Supplementary Tables 2 and 3, respectively. Western blotting performed as described previously [41].

### Cell culture and cell death assays

Cell lines were derived from frozen single cell suspensions of lymphoma-burdened spleens taken from *Eµ-Myc*^*T/+*^*;Bcl-w*^*+/+*^ (wild-type), *Eµ-Myc*^*T/+*^*;Bcl-w*^*+/−*^ (heterozygous), and *Eµ-Myc*^*T/+*^*;Bcl-w*^*−/−*^ (knockout) mice at ethical endpoint (as described in the supplementary methods). Cell death was measured 24 h after in vitro treatment with S63845 (MCL-1 inhibitor; Active Biochem #A-6044) at 0/1/8/40/100/500 nM or ABT-737 (BCL-2 + BCL-XL + BCL-W inhibitor; Active Biochem #A-1002) at 0/1/200/1000/5000 nM. See supplementary materials for additional information.

### Immunophenotyping of lymphoma cells

Lymphoma cells were immunophenotyped as described in the supplementary materials, using antibodies detailed in Supplementary Table 4.

## Supplementary information

Supplementary Information, Tables and Files

Supplementary Figure 1

Supplementary File 1- complete sample information
