# Supplementary material for: BCL-W makes only minor contributions to MYC-driven lymphoma development
Source: Oncogene. 2023 Aug 11;42(37):2776–81. doi: 10.1038/s41388-023-02804-5 (PMC10491490; doi:10.1038/s41388-023-02804-5)
Supplement: Supplementary file 1 — Supplementary Information, Tables and Files [file 41388_2023_2804_MOESM1_ESM.docx]

**Supplementary Materials and methods**

**Husbandry, tumour-identification, and sacrificial processes**

Male *Eµ-Myc^T/+^; Bcl-w^+/-^* mice were crossed with female *Bcl-w^+/-^*_­_ or *Bcl-w^-/-^* mice and offspring were genotyped for *Bcl-w* (primer sequences located in Supplementary Table 1). Mice containing the *Eµ-Myc* transgene were monitored for lymphoma (enlarged spleen/lymph nodes, difficulty breathing, hunched, hind limb paralysis, weight loss) and sick mice were sacrificed at the predetermined humane endpoint as assessed by an experienced animal technician who was blinded to the genotype of the mice. At the point of sacrifice, a retro-orbital bleed was taken, and blood cell counts were measured by Advia (Siemens). Mice were euthanised and enlarged, tumour-bearing organs (lymph nodes, spleen, and thymus) were collected, weighed, and frozen as single cell suspensions generated by mashing tissues through a 100 µM strainer. Survival curves, blood content, and organ weight data were statistically analysed using Prism (GraphPad). Complete mouse cohort population data are available in Supplementary File 1.

**Cell line maintenance, cell death assay processing**

After thawing, cells were spun down and resuspended in fresh FMA medium and plated into 24 well plates to allow outgrowth of *Eµ-Myc* lymphoma cells. *Eµ-Myc* lymphoma cell lines were passaged until they could be maintained at >70% viability.

Cell death assays were set up by plating 3x10^4^ lymphoma cells (in duplicate) into 96 well flat bottom plates, immediately treated with drugs at the indicated concentrations, and then incubated for 24 hrs. To process the assays, cells were stained with Annexin V APC (1:2000, made in house) and propidium iodide (PI, 1 µg/mL, Sigma-Aldrich #P4170), and analysed using an LSR II W flow cytometer and FlowJo software (BD Biosciences). Live cells were identified as Annexin V/PI double negative. Graphs were plotted and statistical analyses performed in Prism (GraphPad).

**Immunophenotyping process**

Frozen tumour single cell suspensions (from spleen or lymph nodes) were thawed into FACS buffer (PBS containing 5% FBS), spun down at 1,500 rpm for 5 min, and resuspended in FACS buffer. 2x10^6^ cells were transferred into 96 well round bottom plates. Plates were spun down at 1,500 rpm for 5 min and supernatant discarded. Cells were resuspended in 50 µL FACS buffer with 10% 24G2 hybridoma supernatant containing rat monoclonal antibodies against Fcγ receptor (made in house) to block non-specific binding of the fluorochrome-conjugated rat monoclonal antibodies (Supplementary Table 4). Cells were incubated with these antibodies for 45 min on ice in the dark. 200 µL of FACS buffer was added to each well and plates were spun down and supernatant discarded. This wash step was repeated, and then cells were resuspended in FACS buffer containing PI (0.5 µg/mL) to exclude dead cells for analysis by flow cytometry using a Fortessa X20 analyser and FlowJo software (BD Biosciences).

**Supplementary Table 1. Primers used for mouse genotyping.** Note that the forward primer, whether genotyping wildtype or knockout *Bcl-w* animals, is identical.

| **Target** | **Forward (5’-3’)** | **Reverse (5’-3’)** |
| --- | --- | --- |
| *Eµ-Myc* transgene | CAGCTGGCGTAATAGCGAAGAG | CTGTGACTGGTGAGTACTCAACC |
| *Bcl-w* (wildtype) | CATACAGCTGGTGCTGTCGTA | GTCTCAAACTCGTCTCCAGCA |
| *Bcl-w* (knockout) | CATACAGCTGGTGCTGTCGTA | CGACGGTATCGATAAGCTTGA |

**Supplementary Table 2. Western blotting primary antibodies.** Primary antibodies used in western blotting in this study.

| **Antigen** | **Host species** | **Dilution** | **Source** |
| --- | --- | --- | --- |
| HSP70 | Mouse | 1:10,000 | Dr Robin Anderson, Olivia Newton-John Cancer Research Institute |
| BCL-W | Rabbit | 1:500 | Cell Signaling Technologies #2724 |
| BCL-XL | Rat | 1:1000 | WEHI mAb facility, clone #9C9 |
| BCL-2 | Mouse | 1:1000 | BD Biosciences #610539 |
| MCL-1 | Rat | 1:2000 | WEHI mAb facility, clone #19C4-15 |
| BIM | Rabbit | 1:1000 | Enzo Life Sciences #ADI-AAP-330-E |
| TRP53 | Rabbit | 1:2000 | Novocastra #NCL-p53-CM5p |
| P19ARF | Rabbit | 1:1000 | Sigma-Aldrich #PC435 |
| MYC | Rabbit | 1:1000 | Cell Signaling Technologies #5605 |

**Supplementary Table 3. Western blotting secondary antibodies.** HRP-conjugated secondary antibodies used in western blotting in this study.

| **Antigen** | **Host species** | **Dilution** | **Source** |
| --- | --- | --- | --- |
| Mouse IgG | Goat | 1:2000 | Southern Biotech #1010-05 |
| Rabbit IgG | Goat | 1.25:2000 | Southern Biotech #4010-05 |
| Rat IgG | Goat | 1:5000 | Southern Biotech #3010-05 |

**Supplementary Table 4. FACS antibodies.** Fluorochrome-conjugated antibodies used in this study.

| **Antigen** | **Clone-Fluorophore** | **Dilution** | **Source** |
| --- | --- | --- | --- |
| B220 | RA3-6B2-BV650 | 1:200 | BioLegend #103241 |
| CD19 | 1D3-A700 | 1:400 | made in house |
| IgM | 5.1-FITC | 1:400 | made in house |
| IgD | 11-26c.2a-BV510 | 1:400 | BD Biosciences #563110 |

**Supplementary Figure 1. Complete western blot images.**

Each column of blots was quantified relative to the HSP70 blot at the top of the column, as they were the same blot, stripped and reprobed as needed. The three groups of columns correspond to the three groups of blots shown in Figure 1E.

**Supplementary File 1. Complete mouse cohort data.** Complete data from the mouse cohort used for all analyses undertaken in this study.
