# Supplementary figures and images for: BCL-W makes only minor contributions to MYC-driven lymphoma development
Source: Oncogene. 2023 Aug 11;42(37):2776–81. doi: 10.1038/s41388-023-02804-5 (PMC10491490; doi:10.1038/s41388-023-02804-5)

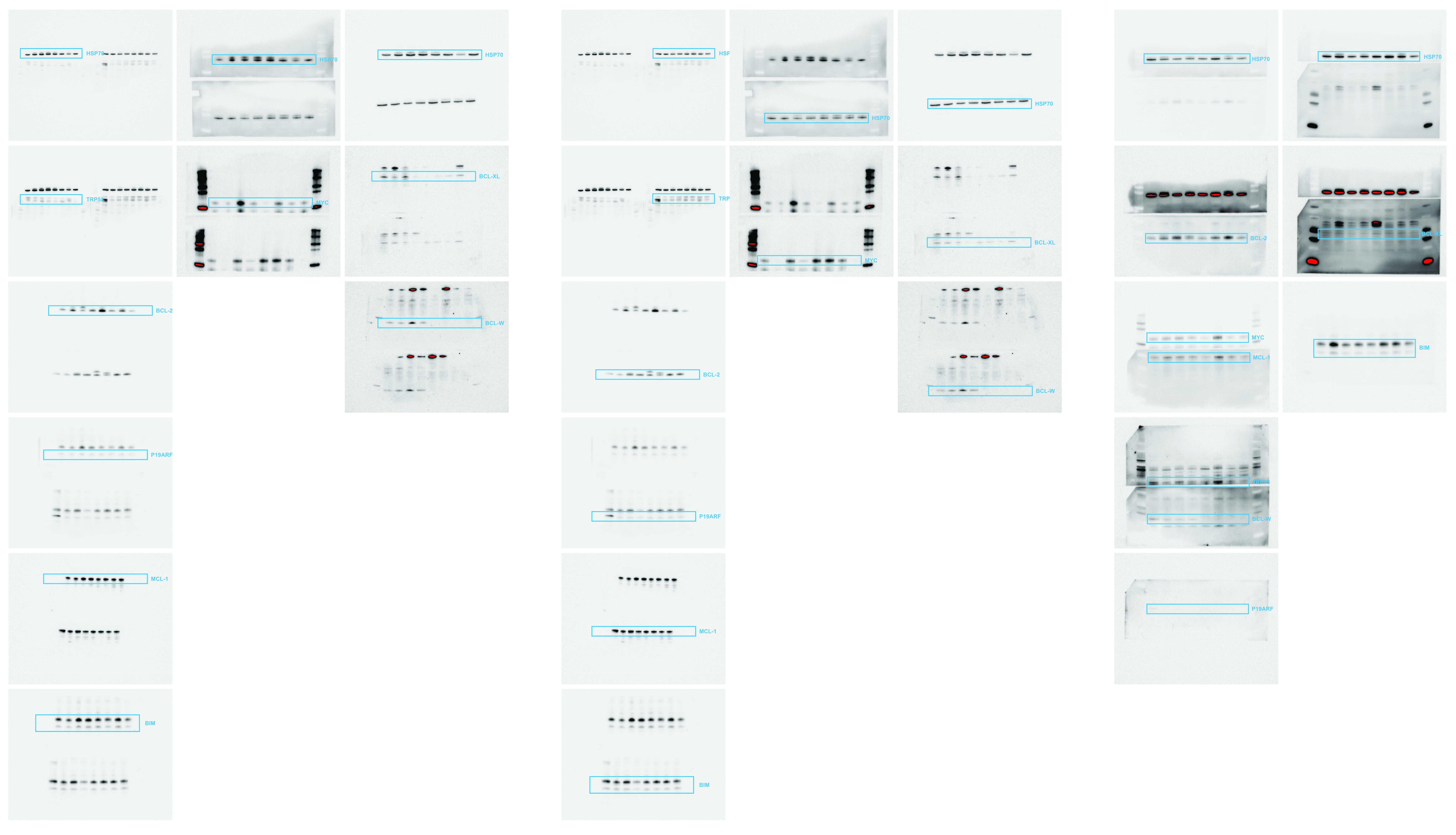

Supplement: Supplementary file 2 — Supplementary Figure 1 [file 41388_2023_2804_MOESM2_ESM.tif]
