# Supplementary material for: BCL-W makes only minor contributions to MYC-driven lymphoma development
Source: Oncogene. 2023 Aug 11;42(37):2776–81. doi: 10.1038/s41388-023-02804-5 (PMC10491490; doi:10.1038/s41388-023-02804-5)
Supplement: Supplementary file 3 — Supplementary File 1- complete sample information [file 41388_2023_2804_MOESM3_ESM.pdf]

								Organ Weights (g)			Advia Results			Tissues Collected		Tissue Used For:		
Animal #	Animal ID	Sex	*Bcl-w*	*Eu-Myc*	Date of Birth	Date of Death	Age in Days (as of 10/03/2023)	Spleen	Thymus	Lymph Nodes	WBC(B) (x10^3 cells/uL)	RBC (x10^6 cells/uL)	Platelets (x10^9 cells/L)	Frozen	Formalin	Cell Lines	Western Blot	FACS
9	4433105	Female	+/-	T/+	27/09/2019	28/11/2019	62	0.75	0.23	1.31	50.82	8.3	447	Y	Y	Y		Y
10	4433106	Female	+/-	T/+	27/09/2019	13/01/2020	108	0.57	1.2	1.09	129.27	9.61	914	Y	Y	Y		Y
44	4476780	Female	+/-	T/+	30/11/2019	07/12/2020	373	0.06	0.04	0.008	3.92	10.81	1744	Y	Y			Y
47	4476783	Male	+/-	T/+	30/11/2019	06/02/2020	68	0.54	0.25	0.53	81.02	9.26	1098	Y	Y	Y		Y
75	4500254	Female	+/-	T/+	08/01/2020	28/07/2021	567				10.41	6.57	190					
84	4508904	Female	+/-	T/+	27/01/2020	09/04/2020	73											
88	4508908	Male	+/-	T/+	27/01/2020	13/04/2020	77											
89	4508909	Male	+/-	T/+	27/01/2020	18/05/2020	112				30.59	8.64	1220					
91	4510252	Female	+/-	T/+	28/01/2020	06/09/2020	222											
93	4510254	Female	+/-	T/+	28/01/2020	11/05/2020	104				57.7	8.59	951					
94	4510255	Female	-/-	T/+	28/01/2020	21/07/2021	540											
96	4510257	Male	-/-	T/+	28/01/2020	11/05/2020	104				80.37	9.43	752					
97	4515014	Female	+/-	T/+	04/02/2020	25/05/2020	111				168.74	7.88	496					
105	4526869	Female	+/-	T/+	22/02/2020	04/05/2020	72											
106	4526870	Female	+/-	T/+	22/02/2020	25/05/2020	93				204.1	9.96	604					
107	4526871	Female	-/-	T/+	22/02/2020	29/06/2020	128	0.31	1.3	0.23	37.02	8.58	180	Y	Y		Y	Y
133	4537993	Male	-/-	T/+	12/03/2020	01/06/2020	81				10.76	9.55	1018					
136	4545439	Male	+/+	T/+	25/03/2020	17/06/2020	84				130.23	10.17	831					
146	4565197	Female	+/-	T/+	07/05/2020	10/08/2020	95				150.46	8.58	396					
149	4570698	Female	-/-	T/+	17/05/2020	05/10/2020	141	1.48	0.13	0.1	32.21	7.76	284	Y	Y	Y	Y	Y
150	4570699	Female	+/-	T/+	17/05/2020	31/05/2021	379	0.4974	0.2472	0.2342	25.84	2.72	114	Y	Y			
151	4570700	Female	+/-	T/+	17/05/2020	17/08/2020	92											
153	4570702	Male	-/-	T/+	17/05/2020	12/08/2020	87											
155	4583650	Female	-/-	T/+	01/06/2020	10/08/2020	70				28.8	8.12	776					
156	4583651	Female	+/-	T/+	01/06/2020	14/09/2020	105				63.9	10.47	829					
158	4583653	Male	-/-	T/+	01/06/2020	28/08/2020	88	0.18	0.93	0.09	85.04	7.87	272	Y	Y	Y	Y	Y
160	4587338	Male	+/-	T/+	16/06/2020	17/09/2020	93				19.15	9.12	810					
161	4591582	Female	-/-	T/+	25/06/2020	20/10/2020	117	0.23	0.99	2.09	14.15	10.17	1074	Y	Y			
162	4591583	Female	+/-	T/+	25/06/2020	21/09/2020	88				377.03	7.51	266					
164	4591585	Female	+/-	T/+	25/06/2020	09/09/2020	76											
166	4591587	Male	-/-	T/+	25/06/2020	14/09/2020	81	0.333	1.778	0.148	50.14	10.78	801			Y	Y	Y
168	4595036	Female	+/-	T/+	01/07/2020	09/11/2020	131				81.44	10.95	565					
171	4595039	Female	+/-	T/+	01/07/2020	21/09/2020	82				88.06	9.92	545					
178	4600810	Female	+/-	T/+	13/07/2020	07/06/2021	329	0.509	0.1548	0.3384	43.61	4.15	281	Y	Y	Y		Y
180	4600812	Male	-/-	T/+	13/07/2020	14/12/2020	154	0.06	0.03	0.01	7.98	14.61	1766	Y	Y		Y	Y
184	4603693	Female	+/-	T/+	17/07/2020	21/09/2020	66				67.54	7.84	648					
185	4606958	Female	+/-	T/+	23/07/2020	28/10/2020	97											
187	4606960	Female	+/+	T/+	23/07/2020	23/11/2020	123				20.58	10.23	837					
188	4606961	Female	+/-	T/+	23/07/2020	28/01/2021	189	0.476	0.319	1.58	62.68	9.3	943	Y				
189	4606962	Male	-/-	T/+	23/07/2020	16/04/2021	267											
191	4606964	Male	-/-	T/+	23/07/2020	02/11/2021	467				17.13	10.46	1236					
192	4614183	Female	-/-	T/+	05/08/2020	28/10/2020	84				116.17	7.77	707					
193	4614184	Female	+/-	T/+	05/08/2020	09/11/2020	96				33.49	8.77	896					
194	4614185	Female	+/-	T/+	05/08/2020	07/12/2020	124	0.51	0.12	1.3	77.59	10.49	1116	Y	Y			Y
198	4622121	Male	+/+	T/+	19/08/2020	30/10/2020	72	0.6848	0.588	0.3116	23.43	9.22	871	Y	Y	Y	Y	Y
200	4624976	Female	+/-	T/+	24/08/2020	23/11/2020	91				50.23	8.62	843					
206	4626788	Female	-/-	T/+	26/08/2020	03/08/2021	342	0.543	0.365	0.523	17.94	8.55	591	Y	Y			
208	4626790	Female	-/-	T/+	26/08/2020	02/11/2020	68				19.6	8.97	691			Y	Y	Y
214	4647829	Female	+/-	T/+	29/09/2020	09/12/2020	71	0.49	0.23	0.43	20.51	9.78	820	Y	Y			Y
215	4647830	Female	+/-	T/+	29/09/2020	18/01/2021	111											
218	4647833	Female	+/-	T/+	29/09/2020	28/12/2020	90											
223	4653944	Female	+/+	T/+	08/10/2020	27/01/2021	111	0.153	0.859	0.017	98.68	9.56	1001	Y			Y	Y
225	4653946	Male	+/+	T/+	08/10/2020	14/12/2020	67	0.17	0.7	0.01	7.41	14.28	1114	Y	Y	Y	Y	Y
229	4653951	Male	-/-	T/+	08/10/2020	09/04/2021	183	0.36	0.12	0.64	19.6	9.47	812	Y			Y	Y
231	4657644	Male	+/-	T/+	14/10/2020	18/12/2020	65											
233	4660595	Female	+/+	T/+	18/10/2020	27/01/2021	101	0.152	0.048	0.025	18.22	12.4	576	Y		Y	Y	Y
245	4684570	Female	-/-	T/+	23/11/2020	31/03/2021	128	0.33	1.83	0.47	7.23	7.3	958	Y			Y	
246	4684571	Female	+/-	T/+	23/11/2020	27/01/2021	65	0.872	0.13	0.191	40.28	9.94	711	Y				Y
248	4684573	Male	-/-	T/+	23/11/2020	27/04/2021	155	0.405	0.4	0.118	155.72	8.2	590	Y			Y	Y
249	4684574	Male	-/-	T/+	23/11/2020	08/03/2021	105	0.3553	0.2583	0.2668	227.48	8.79	689	Y				
251	4689442	Female	+/-	T/+	29/11/2020	08/03/2021	99	0.5311	0.8213	0.391	95.45	8.18	854	Y				
252	4689443	Female	+/-	T/+	29/11/2020	23/02/2021	86	0.45	0.33	1.14	63.18	8.86	764	Y				
259	4694440	Female	+/-	T/+	07/12/2020	26/03/2021	109				106.3	8.47	781					
262	4694443	Female	+/-	T/+	07/12/2020	17/02/2021	72				14.82	10.74	583					
268	4711539	Female	-/-	T/+	04/01/2021	18/05/2022	499	0.1887	0.2083		6.47	8.44	656					
269	4711540	Female	-/-	T/+	04/01/2021	28/04/2021	114				35.09	8.71	251					
274	4713009	Female	+/-	T/+	06/01/2021	22/04/2021	106	0.24	0.71	0.24	22.49	10.54	658	Y				Y
278	4713013	Male	-/-	T/+	06/01/2021	24/05/2021	138				23.4	10.65	878					
282	4713033	Male	+/-	T/+	06/01/2021	09/04/2021	93	0.13	0.78	0.02	36.05	9.86	981	Y		Y		Y
284	4713035	Male	+/-	T/+	06/01/2021	19/04/2021	103				12.19	11.85	1358					
285	4716863	Female	+/-	T/+	10/01/2021	26/03/2021	75				12.17	10.13	861					
294	4734612	Female	+/-	T/+	09/02/2021	14/05/2021	94				141.96	7.85	765					
296	4734614	Female	-/-	T/+	09/02/2021	15/06/2021	126				27.16	8.68	352					
297	4734615	Female	-/-	T/+	09/02/2021	28/05/2021	108				15.21	8.63	1022					
303	4742544	Female	-/-	T/+	22/02/2021	25/05/2021	92	0.6451	0.2039	0.1143	445.35	6.16	329	Y			Y	Y
307	4742548	Male	-/-	T/+	22/02/2021	02/07/2021	130	0.3211	0.4348	0.7834	115.84	8.33	994				Y	
308	4742549	Male	-/-	T/+	22/02/2021	09/06/2021	107				31.56	9	456					
309	4742550	Male	-/-	T/+	22/02/2021	18/05/2022	450	0.137	0.5249		10.48	9.12	1336					
310	4743946	Female	+/+	T/+	24/02/2021	19/05/2021	84	0.792	0.2866	0.1422	345.81	6.48	363	Y		Y	Y	Y
313	4743949	Female	+/+	T/+	24/02/2021	11/06/2021	107	0.4111	0.3783	0.181	227.87	7.76	674	Y			Y	
314	4743950	Female	+/+	T/+	24/02/2021	14/07/2021	140											
318	4743954	Female	-/-	T/+	24/02/2021	24/12/2021	303											
322	4743958	Female	-/-	T/+	24/02/2021	19/05/2021	84	0.6915	0.4483	0.8596	114.49	7.98	542	Y				
324	4743960	Female	-/-	T/+	24/02/2021	17/05/2021	82	0.2	0.04	0.142	39.5	8.43	705	Y		Y	Y	Y
325	4743961	Female	-/-	T/+	24/02/2021	11/03/2022	380											
329	4751635	Female	+/+	T/+	08/03/2021	11/06/2021	95	0.4789	0.5089	0.2263	59.01	8.72	720	Y		Y	Y	Y
334	4772701	Female	+/-	T/+	08/04/2021	25/08/2021	139											
337	4772704	Female	+/-	T/+	08/04/2021	18/06/2021	71				137.25	6.55	652					
340	4772696	Female	+/+	T/+	08/04/2021	16/08/2021	130	0.5441	0.7424	1.854	34.26	9.54	806	Y			Y	
345	4793110	Female	+/+	T/+	09/05/2021	16/08/2021	99	0.3518	0.9891	0.2861	311.29	7.96	772	Y			Y	
346	4793111	Male	+/+	T/+	09/05/2021	24/06/2021	46	0.1437	0.1971	0.5565	8.16	10.23	651	Y			Y	
352	4821336	Female	+/+	T/+	21/06/2021	01/10/2021	102	0.3403	1.333	0.0332	109.05	10.43	757				Y	Y
355	4821339	Female	+/+	T/+	21/06/2021	17/09/2021	88				87.15	6.38	1780					
360	4839784	Female	+/+	T/+	19/07/2021	27/05/2022	312	1.0422	0.0505	0.0129	193.69	7.09	326					
361	4839785	Female	+/-	T/+	19/07/2021	9/1/2023	539											
367	4854681	Female	+/+	T/+	10/08/2021		577											
386	4898987	Female	+/-	T/+	18/10/2021	21/01/2022	95				23.08	9.65	959					
388	4898989	Female	+/-	T/+	18/10/2021	25/11/2022	403				36.86	11.4	847					
396	4919149	Female	+/+	T/+	18/11/2021	18/02/2022	92	0.397	1.3244	0.0923							Y	Y
402	4926328	Female	+/+	T/+	29/11/2021	21/01/2022	53				9.21	11.6	441					
403	4926329	Female	+/-	T/+	29/11/2021	01/04/2022	123				42.97	10.01	878					
405	4928723	Female	+/-	T/+	03/12/2021	15/02/2022	74											
406	4928724	Male	+/-	T/+	03/12/2021	12/12/2022	374											
409	4928727	Male	+/+	T/+	03/12/2021	02/02/2022	61											
432	4966104	Female	-/-	T/+	07/02/2022	30/12/2022	326											
433	4966105	Female	-/-	T/+	07/02/2022	7/10/2022	242				3.34	3.75	930					
435	4966107	Female	-/-	T/+	07/02/2022	24/06/2022	137				205.06	6.83	236					
436	4966108	Male	+/+	T/+	07/02/2022	18/05/2022	100	0.9709	0.4245	0.7215	125.12	9.56	210					Y
440	4966112	Male	+/+	T/+	07/02/2022	01/07/2022	144	0.4204	0.2934	0.5215	193.13	8.34	743					
444	4971085	Female	+/-	T/+	17/02/2022	22/04/2022	64											
446	4971087	Female	+/-	T/+	17/02/2022	12/05/2022	84	0.6085	0.0965	0.4684	21.89	9.83	874					
472	4990985	Male	+/-	T/+	22/03/2022	27/2/2023	342											
473	4990986	Male	+/-	T/+	22/03/2022		353											
476	4993844	Male	+/-	T/+	29/03/2022		346											
477	4993845	Male	+/-	T/+	29/03/2022	4/11/2022	220				57.19	9.52	691					
478	4993846	Male	+/-	T/+	29/03/2022	29/11/2022	245				37.61	7.75	873					
479	4993847	Male	+/-	T/+	29/03/2022		346											
480	4993848	Male	+/-	T/+	29/03/2022	30/12/2022	276											
481	4993849	Male	+/-	T/+	29/03/2022	15/7/2022	108				13.42	9.66	1089					
484	4996587	Female	+/+	T/+	02/04/2022	14/06/2022	73	0.498	0.251	0.15	64.61	9.14	603					
487	4996590	Male	+/+	T/+	02/04/2022	24/06/2022	83	0.5479	0.4382	0.349	143.79	9.21	619					Y
488	4993851	Female	+/-	T/+	29/03/2022	13/7/2022	106				11.7	10.64	919					
492	4993855	Male	+/+	T/+	29/03/2022	20/1/2023	307											
499	5012216	Male	+/+	T/+	01/05/2022	15/8/2022	106	0.516	0.221	0.13	36.25	9.06	710					
500	5012217	Male	+/-	T/+	01/05/2022	24/06/2022	54	0.5253	0.1102	0.9635	11.66	8.43	694					
502	5019258	Female	+/+	T/+	01/05/2022		313											
518	5031680	Female	+/+	T/+	03/06/2022	5/10/2022	124				7.93	11.3	612					
525	5043885	Female	+/+	T/+	24/06/2022	04/10/2022	102				23.5	10.65	1052					
526	5043886	Female	+/-	T/+	24/06/2022		259											
529	5043889	Male	-/-	T/+	24/06/2022	21/10/2022	119				42.31	9.62	746					
530	5043890	Male	-/-	T/+	24/06/2022		259											
533	5062296	Female	+/-	T/+	27/07/2022	05/10/2022	70				6.38	8.97	263					
535	5062298	Female	+/-	T/+	27/07/2022	05/10/2022	70				195.2	8.39	879					
536	5062299	Female	+/-	T/+	27/07/2022	18/11/2022	114				107.46	10.57	635					
538	5062301	Male	+/-	T/+	27/07/2022	11/10/2022	76											
541	5068783	Female	+/-	T/+	08/08/2022		214											
546	5081520	Female	+/-	T/+	27/08/2022		195											
551	5102431	Female	+/+	T/+	02/10/2022		159											
553	5102433	Female	+/-	T/+	02/10/2022	24/02/2023	145											
556	5107693	Female	+/-	T/+	11/10/2022	20/02/2023	132											
558	5107695	Female	+/-	T/+	11/10/2022	07/12/2022	57				16.4	9.59	537					
559	5107696	Female	-/-	T/+	11/10/2022		150											
560	5107697	Female	+/-	T/+	11/10/2022		150											
565	5107704	Female	+/+	T/+	11/10/2022	25/01/2023	106											
566	5107705	Female	+/-	T/+	11/10/2022		150											
567	5107706	Male	+/+	T/+	11/10/2022	24/02/2023	136											
572	5132651	Female	-/-	T/+	21/10/2022	20/02/2023	91											
576	5132655	Female	+/-	T/+	21/11/2022	23/01/2023	63											
578	5132657	Male	+/+	T/+	21/11/2022	13/03/2023	112	0.071	0.0773	0.0258	7.57	11.27	1357					
